# Disparities in Childhood Obesity Prevalence and Spatial Clustering Related to Socioeconomic Factors in Isaan, Thailand

**DOI:** 10.3390/ijerph20010626

**Published:** 2022-12-29

**Authors:** Hiranya Sritart, Somchat Taertulakarn, Hiroyuki Miyazaki

**Affiliations:** 1 Faculty of Allied Health Sciences, Thammasat University, Pathum Thani 12120, Thailand; 2 Center for Spatial Information Science, University of Tokyo, Chiba 277-8568, Japan

**Keywords:** childhood obesity, socioeconomic factor, spatial analysis, GIS

## Abstract

Globally, rapid economic growth has contributed to an overall increase in the incidence of childhood obesity. Although the prevalence of obesity has been well recognized, the disparities related to a region’s socioeconomic environment in terms of the incidence of obesity are still less understood. Therefore, the purpose of this study was to examine the spatial pattern of childhood obesity and identify the potential associations between childhood obesity and socioeconomic environment in the northeastern region of Thailand, Isaan. Using nationally collected obesity data from children aged 0–5 years in 2019, we employed a geographic information system (GIS) to perform obesity cluster analysis at the smaller regional level, investigating a total of 322 districts in study area. Global and local statistical approaches were applied to calculate spatial associations between the socioeconomic status of neighborhoods and childhood obesity. The study revealed that 12.42% of the total area showed significant clusters at the district level, with high values observed in the western and northeastern areas. The results of the spatial statistical model revealed that childhood obesity was significantly positively associated with areas exhibiting high levels of socioeconomic environment factors. Identifying the associated factors and highlighting geographic regions with significant spatial clusters is a powerful approach towards understanding the role of location and expanding the knowledge on the factors contributing to childhood obesity. Our findings, as a first step, offer valuable references that could support policy-makers and local authorities in enhancing policy development with the aim of reducing childhood obesity and improving public health.

## 1. Introduction

Childhood obesity is one of the most serious worldwide public health challenges of the 21st century [1]. Over the past 40 years, the global incidence of obesity among school-aged children and adolescents has risen dramatically from just 4% in 1975 to more than 18% in 2016 [2]. According to the WHO, more than 340 million children and adolescents aged from 5 to 19 were overweight or obese, whereas an estimated 38.2 million children under the age of five were overweight or obese, in 2019 [2]. The epidemic has escalated and has affected every country, particularly in many low- and middle-income countries due to their urban settings [3,4,5]. Rapid economic development in these regions has resulted in changes in nutrition, as well as physical and social activities and behaviors, which have contributed to the rapid spread of the obesity epidemic over the last few decades [3]. Many Asian countries have undergone socio-economic and lifestyle transitions due to globalization and rapid urbanization [6,7]. In Thailand, the prevalence of childhood obesity has also significantly increased in the last two decades. The percentages of overweight and obese children under age 5 almost doubled from 5.8% in 1997 to 9.2% in 2019 [8,9]. Studies have also revealed that overweight and obese children have a higher risk of gaining weight in adulthood, with a high risk of obesity-related comorbidities [10,11,12,13]. This problem has become a global public health issue, with interventions involving all institutions [14].

Due to its significant impact on health outcomes and the increasing prevalence of childhood obesity, several studies have aimed to understand this phenomenon from various perspectives. Physiologically, obesity is caused by an energy imbalance in which caloric intake exceeds the energy required by metabolism [15]. Apart from genetic and psychological factors, researchers have discovered that environmental, socioeconomic, and behavioral factors influence the prevalence of obesity. Studies show that factors related to the built environment, such as low population density, poor street connectivity, and a lack of sidewalks, are associated with decreased physical activity and an increased risk of being overweight [16,17]. The findings of previous studies have also revealed that geographical areas with socioeconomic disparities and environmental factors may also contribute to the development of an obesogenic environment for children [18,19]. Food intake has been recognized as a complex multifactorial form of behavior, in which personal and environmental factors interact to influence what children consume, and several researchers have observed that one’s socioeconomic environment affects human behavior, contributing to the development of obesity [19,20,21]. The availability of economic, social, and physical resources has been proposed to influence childhood outcomes through collective socialization [19]. For example, parks, schools, supermarkets, and community organizations were less common in disadvantaged neighborhoods, and therefore this lack of infrastructure had the potential to weaken collective life by cultivating negative social capital through adult modeling and peer approval of unhealthy behaviors [20,21]. Furthermore, advances in technology that have expanded the home environment, which have been found to be more common in inner-city neighborhoods, might lead to a decline in energy expenditure and an increase in secondary behaviors such as playing video games and watching TV [22,23].

In recent years, there has been an increase in the amount of studies examining the relationship between neighborhood environmental factors and socioeconomic indicators [24]. Several studies have been conducted to determine the factors associated with obesity risk. Socioeconomic status (SES), race, and birth weight were reported to be associated with overweight or obese kindergarten-aged children [25]. Disparities in educational level and low income have also been reported to be related to a higher prevalence of obesity [26,27]. However, the relationship between socioeconomic status and childhood obesity varies by country, depending on the country’s socioeconomic standing [28,29]. Even in high-income countries, various studies have reported an inverse relationship between socioeconomic status and children’s weight status [24,30], whereas other researchers found a positive relationship among children [24,26]. Consequently, due to the inconsistency of the association, it is necessary to investigate what factors affect the extent of the correlation. Therefore, more study cases in different locations are greatly desired, particularly in the middle-income countries such as Thailand with diverse socioeconomic factors. Furthermore, studies often ignore the geographical relevance of neighboring regions, particularly in terms of socioeconomic factors. Little attention has been paid to the spatial patterns of obesity that may result from the neighborhoods in which children live and go to school. Indeed, more research is required to investigate the relationship between neighborhood context and childhood obesity.

A geographic information system (GIS) is a potential tool that can enable us to understand the spatial epidemiology and the incidence of diseases. Several studies in health research have recognized the importance of GISs. This system offers a comprehensive set of tools for exploring geographic patterns of health outcomes and allows the visual representation of geographic data, as well as the recognition of spatial relationships in epidemiological data [31]. Several innovative studies have employed GISs to explore spatial clusters of obesity incidents to emphasize areas for targeted interventions [32,33,34]. However, the majority of previous analyses were carried out using large geographic units, such as countries or provinces. This approach makes it challenging to provide effective local policy implementation or decision-making for obesity interventions.

Regarding the heterogeneity among spatial units and the limited geospatial studies conducted on childhood obesity in Thailand, the purpose of this study was first to investigate the spatial distribution and patterns of childhood obesity that may be associated with the shifting of regional growth in the country. Second, we aimed to examine whether socioeconomic factors were associated with the spatial clustering patterns of childhood obesity. Finally, we aimed to investigate the differential spatial clustering of childhood obesity at the regional level. To the best of the authors’ knowledge, this paper represents the first effort to investigate several factors associated with childhood obesity with a focus on socio-environmental conditions at a smaller geographical scale, exploring spatial inequalities in order to provide information for local government interventions in this area.

## 2. Materials and Methods

### 2.1. Study Area

**Figure 1:**
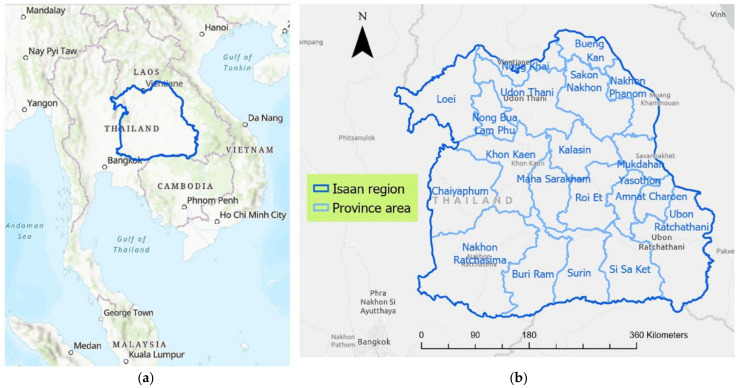
Study area, Isaan region of Thailand: (**a**) location in Northeast Thailand; (**b**) the 20 provinces which make up the region.

The study area examined in this research was the northeastern region of Thailand, Isaan. Geographically, Isaan is Thailand’s largest region, covering an area of 168,854 km^2^, located in Northeast Thailand. The region is surrounded by the Mekong River to the north and the east, along with the Laos–Thailand border and Cambodia in the south, as shown in Figure 1. To the west, the Isaan region is connected to northern and central Thailand. The Isaan region consists of 20 provinces and 322 districts. The region is considered the largest and most populous in the country. According to the National Statistical Office of Thailand, almost one third of the country’s population is located in the Northeast region, which has a population of 18.5 million people [35]. However, this region is Thailand’s poorest region in terms of per capita regional value added, compared to other regions of Thailand, namely, central, southern, and northern Thailand [36]. This region has been experiencing rapid change in recent years, including technological changes, epidemiological transitions in health, and changes in social systems [37,38]. Generally, the Isaan population depends on the agriculture sector, with urban areas accounting for about 15% of the total [39]. Due to the extreme inequality of the economy and growth rates as compared to other regions of Thailand, several studies have revealed that the regional imbalances are becoming more critical in this region, e.g., disparities in healthcare services [40], different patterns of population and density [41], and inequities in the development of infrastructure and regional growth [42].

Due to its rapid socioeconomic transition, Thailand is now confronted with the problem of childhood obesity. Concerning this issue, Isaan is one of the major regions facing a challenging situation, with the largest number of obese children among the population and the highest number of obese children under 5 years old, at 28.45 percent, according to a national report [35]. Several studies have revealed that obesity in childhood has reached alarming proportions [4,43,44].

### 2.2. Collected Data

To ensure an effective investigation of the spatial distribution of childhood obesity and to explore the disparities in SES characteristics in the study area, the data employed in this research were obtained from several resources. The main essential data required for the study were childhood obesity, demographic, and socio-economic data, and data concerning the boundaries of the study area. Table 1 presents detailed information about the data, sources, and applications applied in this study.

This study was conducted using nationally and officially collected data from the Ministry of Public Health, Thailand. Childhood obesity was defined as being overweight compared to the growth graph with a weight-for-height Z-score greater than +2SD, according to the length or height criteria for children aged 0–5 years in the guidelines provided by the Department of Health, Thailand, which were adapted from the WHO child growth standards [45]. These childhood obesity data were downloaded from the Health Data Center (HDC) (https://hdcservice.moph.go.th/, accessed on 31 January 2022). The data were collected as the percentage of children aged under 5 years old with obesity in each district in the year 2019 for every province of in study area and these were compiled in the study.

The socio-economic data that were applied in this study consisted of population data, household data, mean annual income, percentages of adults with higher education, unemployment, and immigrant data. These data were collected from the Community Development Department, Thailand (https://ebmn.cdd.go.th/, accessed on 2 April 2022). To investigate the spatial distribution of these data at a relatively small scale, district-level SES data were used in this study.

The boundaries and geographic data applied in this study were obtained from the official Humanitarian Data Exchange (HDX), which is an open data platform managed by the United Nations Office for the Coordination of Humanitarian Affairs (OCHA, New York, NY, USA), which provides a database of political-administrative boundaries for research. The GIS boundary data for the study area were gathered on the administrative scale of provinces and smaller district levels. The data were collected in a shape-file format and utilized as the basis for further processing.

### 2.3. Methodology

#### 2.3.1. Data Visualization and the Spatial Distribution of Childhood Obesity

In order to investigate childhood obesity and the disparities in the existing environment for further spatial data analysis, the area-based mapping method was applied in aggregating information and visualizing the spatial distribution of childhood obesity. In this study, we applied the original administrative unit of the province level and the smaller-area resolution of the district level for the study area to address local interventions for further analysis. The geographical borders of districts were represented in a polygon format in the GIS system, where each polygon represented one district and had a unique ID number within the attribute database. Then, the rates of childhood obesity and overweight were joined by means of the same attributes at the district level in the study area. According to the previous literature, to process and normalize disease values in terms of spatial epidemiology, the obesity index used in this study was calculated and defined as childhood obese/overweight per 1000 observations [46]. The SES variable data explored in this research were also aggregated with the same district codes in the GIS environment. In this study, ArcGIS 10.7.1 (ESRI, Redlands, CA, USA) was used to process the data and map the spatial distribution of childhood obesity.

#### 2.3.2. Spatial Distribution and Clustering of Childhood Obesity

To investigate the spatial epidemiology of childhood obesity across the Isaan region of Thailand, the global Moran I statistic was applied to assess whether the spatial distribution of childhood obesity was dispersed, clustered, or randomly distributed. The global Moran I is a method used to measure spatial dependence (spatial autocorrelation) based on locations and childhood obesity data simultaneously. In our study, the null hypothesis of the Moran I analysis was that the spatial pattern in the prevalence of childhood obesity across Isaan area was random. The mathematical equation of Moran’s I is as follows:

(1) $$I=\frac{n}{S_{0}}\frac{\sum_{i=1}^{n}\sum_{j=1}^{n}w_{\mathrm{ij}}\left(x_{i}-\bar{x}\right)\left(x_{j}-\bar{x}\right)}{\sum_{i=1}^{n}{\left(x_{i}-\bar{x}\right)}^{2}},$$

where I is the global Moran I statistic for spatial autocorrelation in the study. n is the number of districts. $x_{i}$ is the individual obesity index, whereas $\bar{x}$ is the mean value of the attribute in the study. $w_{\mathrm{ij}}$ indicates the spatial weights assigned to pairs of district units between observations i and j, whereas i is an individual observation and j refers to observations at other locations. $S_{0}$ is an aggregate of all spatial weights.

The model computes the entire dataset and generates a single output of positive or negative dependence, which indicate that observations made at near spatial distances have similar or distinct values, respectively. The range of the output of Moran’s I is from −1 to +1. A Moran I index close to −1 would indicate that obesity in children aged under 5 years old was dispersed, a Moran I index close to +1 would indicate that childhood obesity across Isaan was clustered, and childhood obesity would be randomly distributed when the value was close to 0. The rejection of the null hypothesis implied a spatial autocorrelation in the prevalence of childhood obesity that indicated the presence of spatial heterogeneity.

To indicate the physical location of childhood clustering, local Moran I analysis was also applied in this study [47]. This analysis enabled us to generate an analytical output for each individual in the dataset to evaluate finer-grained patterns within the research area. Each individual’s local Moran I statistic was computed as follows:

(2) $$I_{i}=\frac{\left(x_{i}-\bar{x}\right)}{\sigma^{2}}\sum_{j=1}^{n}w_{\mathrm{ij}}\left(x_{j}-\bar{x}\right),$$

where $I_{i}$ is the local Moran I statistic for localized spatial autocorrelation. n is the number of districts. $x_{i}$ is the individual obesity index value, whereas $\bar{x}$ is the mean value of the attribute in the study. σ^2^ is the variance of the childhood obesity index, which is the spatial weights assigned to pairs of district units between observations i and j. The output can identify five types of spatial patterns—high–high (HH), high–low (HL), low–high (LH), low–low (LL), and not significant—which can be used to map the study area to visualize the location of identified clusters. In this study, we were particularly interested in the HH and LL patterns that represented regions with high and low obesity indices for children, respectively.

Regarding the analysis of childhood obesity in the study area for each province with a smaller resolution, using districts as the unit of analysis, the average number of districts for each province was first calculated. Then, this parameter referring to the specific number of neighbors was later applied to calculate the global and local Moran I values to analyze the closest features that were included in the spatial clustering analysis.

#### 2.3.3. Spatial Analysis between Childhood Obesity and SES Factors

The global and local Moran I tests provided statistical evidence of spatial patterns for the variable of interest that explained the importance of the geographical influence on childhood obesity. However, the results did not take into consideration the impact of other explanatory variables. Therefore, in order to determine the factors influencing the presence of spatial heterogeneity across the study area, we employed global GLR, generalized linear regression, and a local geographically weighted model for further analysis.

#### 2.3.4. Global Statistical Model of Generalized Linear Regression (GLR)

In this study, we aimed to investigate the associations between childhood obesity and SES factors. Based on previous studies, the following SES variables were applied in this analysis: annual income, population density, number of households, level of education, rate of unemployment, and proportion of immigrants. Spatial regression modeling was performed to identify determinants of the geographical heterogeneity of obesity among children aged less than 5 years old. To evaluate these associations, Poisson regression analysis was applied to explore and explain the relationship between the dependent variable, which was measured as the total number of children with obesity, and the independent variables. To quantify the relationship between the independent variable and the dependent variables, the GLR model was used not only to analyze relationships but also to quantify their direction and strength [48]. Additionally, the model was effective for separating the influence of several variables on the final result.

The general GLR equation is defined as follows:

(3) $$y_{i}={\;\beta }_{0}+{\;\beta }_{1}{\;x}_{1i}+{\;\beta }_{2}{\;x}_{2i}+\ldots +{\;\beta }_{\mathrm{mi}}{\;x}_{\mathrm{mi}}+{\;\varepsilon }_{i},$$

where y is the dependent variable. $x_{\mathrm{mi}}$ indicates the independent variables investigated in this study. $\beta_{\mathrm{mi}}$ represents the regression coefficients that represent the strength and type of the relationships by which the explanatory variables influence the dependent variable. $\varepsilon_{i}$ is the residual of the model, indicating that predicted model is over- or underestimating the observed values.

This model assumes stationarity or a consistent relationship and employs a single equation to calculate the relationship between the dependent and independent variables. The global regression analysis results are reported as β-coefficients and ε-standard errors. All reported *p*-values were compared to a 5% significance threshold.

#### 2.3.5. Local Statistical Model of Geographically Weighted Regression (GWR)

Tobler’s first law of geography states that everything is related to everything else, but near things are more related than distant things. Therefore, in our research we aimed to explore a local model in the analysis of the correlation between childhood obesity and SES factors in the study area. Geographically weighted regression (GWR) was applied in this study for spatial data analysis and to explore spatial relationships.

In a global model, the GLR equation is created for the entire study area; however, in the local model each feature of the study area has its own equation in the GWR. This is the main difference between these models. The GWR equation is calibrated using data from the neighboring features as follows:

(4) $$y_{i}\left(u\right)={\;\beta }_{0i}\left(u\right)+{\;\beta }_{1i}\left(u\right){\;x}_{1i}+{\;\beta }_{2i}\left(u\right){\;x}_{2i}+\ldots +{\;\beta }_{\mathrm{mi}}\left(u\right){\;x}_{\mathrm{mi}},$$

where y is the dependent variable at a location, u, that regressed on a set (m) of independent variables x at the same location. β indicates that the parameter describes a relationship around location u and is specific to that location. Every spatial unit, i, of the area of the study is given its own equation by GWR, which includes both dependent and explanatory variables.

Regarding the investigation of the spatial heterogeneity of the association between SES factors and childhood obesity in the study area, the explanatory variables for the local modeling were chosen based on the outcome of the global study. In the local model, each of the identified SES factors was designated as an explanatory variable, whereas the prevalence of childhood obesity was defined as the dependent variable. Since each province in the Isaan area has a different number of districts, the number of neighboring features calculated in this study was defined based on the average number of districts for each province. The Akaike information criterion (AICc) for the GWR model was also determined. The AICc is a relative goodness-of-fit statistic for comparing GLR models. The model with the smallest AICc provides the closest approximation to reality [49,50].

## 3. Results

### 3.1. Spatial Distribution

Table 2 shows the descriptive statistics for each variable utilized in the study. The obesity index ranged from 34.14 to 230.47, with a mean value of 111.42 per district area. With regard to the SES variable, the average annual income of the population in the Isaan region was lower than 70,000 THB, with the minimum and maximum of 47.61 and 147.64 thousand THB, respectively. The population density ranged from 13 to 297, with an average of 93 people per square kilometer. The proportion of individuals with higher education in the study area varied from 2.83 to 21.36, with an average of 6.20. The highest percentage of unemployed individuals at the district level was lower than 10, as was the proportion of immigrants, with averages of 4.5% and 0.25%, respectively.

**Figure 2:**
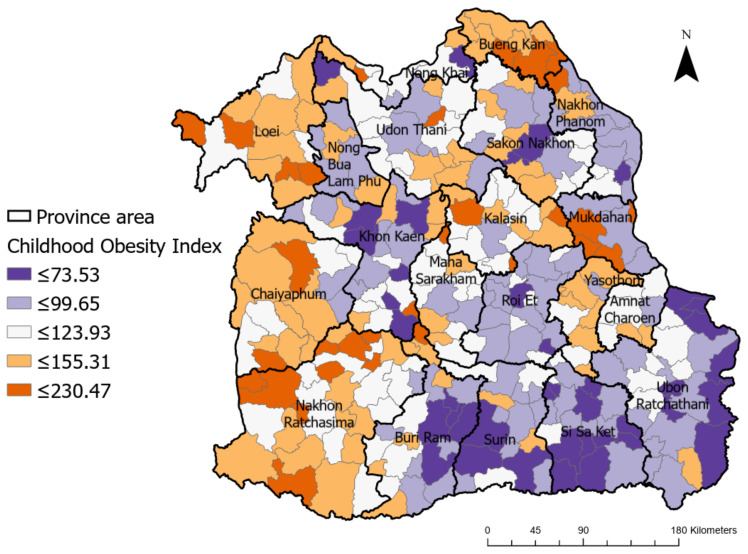
Spatial distribution of the childhood obesity index across the Isaan region of Thailand.

Regarding the spatial distribution of childhood obesity across the Isaan region in Thailand, Figure 2 presents the obesity indices of children aged less than 5 years old in five different clusters which are illustrated in distinct colors ranging from dark purple to dark orange, indicating low and high levels of childhood obesity, respectively. The geographical distribution and clustering of obesity are presented with a smaller resolution than the province level and they showed variations across geographic areas. As can be seen in Figure 2, the regions with high levels of childhood obesity were mostly located in the western part of the study area, e.g., Loei, Chaiyaphoom, and Nakon Ratchasima, whereas regions with very low levels were found in the southeastern part of Isaan, e.g., Surin, Sri Saket, and Ubon Ratchathani. However, to be able to obtain clear findings on the spatial patterns of childhood obesity across the Isaan region, it was necessary to investigate the spatial autocorrelation further.

**Figure 3:**
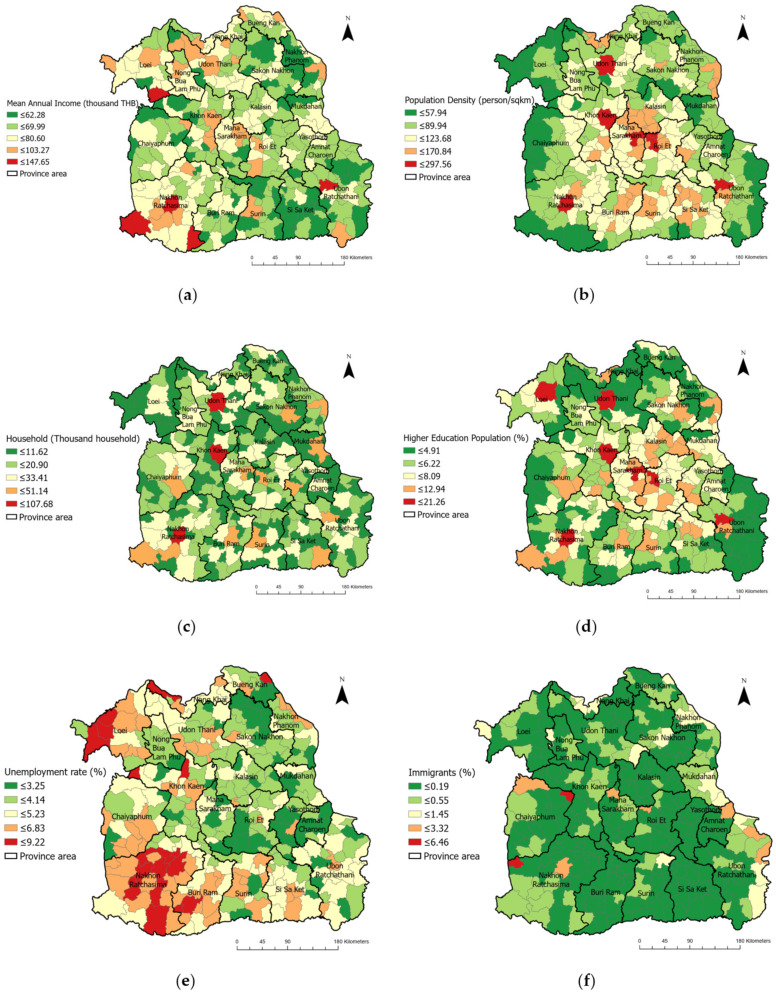
District-level spatial distribution of SES variables across Isaan: (**a**) annual income; (**b**) population density; (**c**) number of households; (**d**) rate of higher education level; (**e**) unemployment rate; (**f**) proportion of immigrants.

As a visualization of the disparity in SES factors in the study area, Figure 3 illustrates the wide range of values of each variable across Isaan, depicting the geographical heterogeneity between districts, which emphasized the spatial inequalities of the region.

### 3.2. Spatial Analysis and Clustering of Childhood Obesity

#### Spatial Autocorrelation and Cluster Identification

To explore the spatial patterns of childhood obesity across Isaan, we investigated whether spatial autocorrelation existed in the study area. As shown in Table 3, the global Moran I results demonstrated that there was a significant, positive global spatial autocorrelation in terms of childhood obesity incidence, with an index value of 0.2855 and a corresponding *p*-value of less than 0.001. This finding implies that the overall spatial pattern of the incidence of childhood obesity in the Northeast region of Thailand was non-random in nature, indicating the presence of spatial heterogeneity and therefore making further analysis essential.

Local Moran I statistics provided further details regarding the different clustering patterns in childhood obesity in the study area. The local Moran I results showed a significant local clustering pattern. The numbers of high–high (HH), high–low (HL), low–high (LH), and low–low (LL) clusters are presented in Table 3. A total of 40 HH and 52 LL statistically significant spatial clusters were observed, representing 12.42% and 16.15%, respectively.

**Figure 4:**
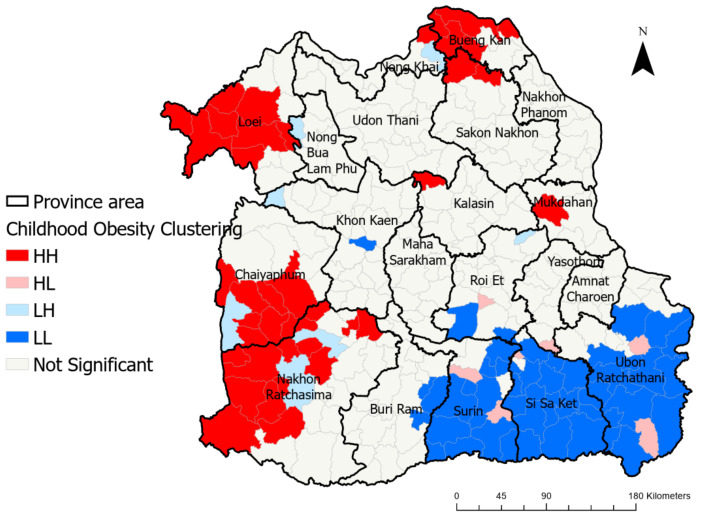
Clusters and outliners of childhood obesity across Isaan.

Furthermore, Figure 4 shows the clusters and outliners of obesity in children aged less than 5 years old across Isaan, representing the results of the local Moran I analysis. Particularly, the map indicates a significant cluster at the district level with a high value (hot spot, high–high), a low-value (cold spot, low–low) and outliners (low–high or high–low). Focusing on the hotspot, HH clusters were found mainly in Loei, Chaiyaphum, Nakon Ratchasima, and Bueng Kan and partially in Kalasin and Mukdahan. The low-density clusters (LL) of childhood obesity were generally detected in southeastern parts of Isaan, particularly Surin, Sri Sa Ket, and Ubon Ratchathani.

### 3.3. Spatial Association of Childhood Obesity Prevalence and SES Factors

#### 3.3.1. Global Relationship between Childhood Obesity and SES Variables

Table 4 summarizes the results of the Poisson regression analysis of the relationship between the SES variables and childhood obesity. According to the GLR analysis conducted across all districts in the Isaan region of Thailand, all SES variables applied in this study were statistically significantly associated with the prevalence of childhood obesity, except the proportion of immigrants. First and foremost, childhood obesity was positively correlated with annual income, percentage of adults with higher education, and percentage of unemployment, with all *p*-values < 0.001. The results also revealed that the prevalence of childhood obesity in Isaan was inversely associated with the density of the population and the number of households. This indicates that, overall, areas with a high annual income, a high number of adults with higher education, and a high rate of unemployment were likely to have a greater prevalence of childhood obesity.

#### 3.3.2. Local Relationship between Childhood Obesity and SES Variables

As previously noted, GWR was only implemented for the SES factors selected based on the confirmed Poisson regression results that required further analysis in terms of their spatial variations across the Isaan region. GWR and the significant SES variables listed in Table 3 were applied to measure the spatial variations of the correlation between childhood obesity and these influencing factors.

Table 5 presents the results of the GLR analysis compared with the GWR analysis, representing the global and local model, respectively. The results obtained for the local statistical model exhibited better quality than those of the global model, as shown by the higher deviance value of the local model, at 0.8844, compared to the global value of 0.1621. This deviance value is a goodness-of-fit metric that quantifies the performance of a local model, and it varies from 0.0 to 1.0, with greater values indicating superior performance. Furthermore, the results of the local statistical model had an Akaike information criterion (AICc) of 1835.8, which was lower than the global model’s AICc (4904.0), which also indicates better performance compared to the global model.

**Figure 5:**
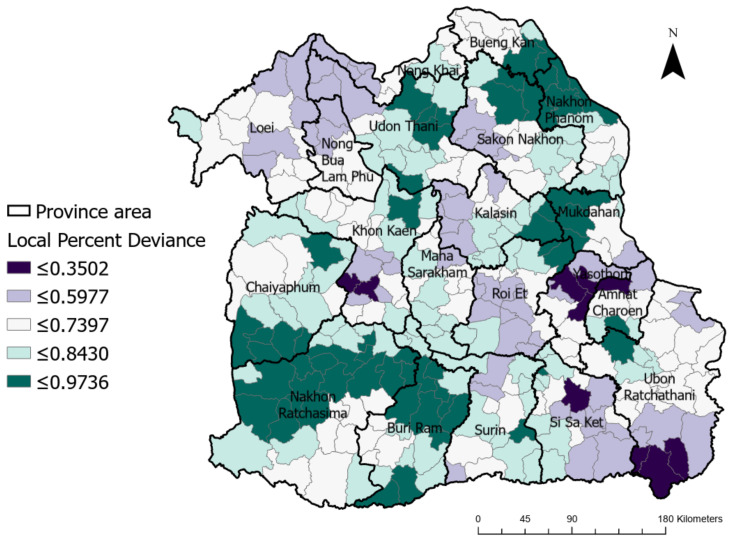
Local percentage deviance based on GWR results across the study area.

Based on the GWR results, Figure 5 presents the local percentages of deviance across the study area, showing that greater fit and higher performance were observed in the range of 0.3502–0.9736, illustrated with colors ranging from dark purple to dark green, respectively.

The mean coefficient values of the factors that were significant in explaining the prevalence of childhood obesity, as identified by GWR, are presented in Table 6. Based on the GWR results shown in Table 6, annual income and the proportion of adults with higher education were positively associated with childhood obesity in the study area. On the other hand, population density, number of households, and percentage of unemployed individuals were negatively associated with childhood obesity in the study area.

In Table 7, the SES variables are summarized and separated for each of the identified spatial clusters of HH, HL, LH, and LL in relation to the prevalence of childhood obesity. In the western and northern parts of Isaan, a high clustering value, high annual income, a high proportion of unemployed individuals, and high proportion of higher-educated adults were positively associated with childhood obesity, whereas population density and the number of households showed a negative contribution to the prevalence of childhood obesity. For the southern and western clusters of low childhood obesity, the proportion of unemployment, population density, and households showed a negative association with the prevalence of childhood obesity. However, in both high, and low clusters in this study area, the percentage of adults with a higher education degree was shown to be the strongest factor.

## 4. Discussion

To the best of our knowledge, this is the first report published on the relationship between childhood obesity and socioeconomic status with a focus on several environmental inequalities in Isaan, Thailand. In this study, we explored the spatial distribution of childhood obesity, analyzing spatial clustering as well as the spatial relationships between the factors influencing the obesity indices of children aged less than 5 years old at the district level throughout the Isaan region of Thailand—a region with geographical disparities. By examining specific geographical patterns and determinants of the observed spatial distribution, these spatial analyses have expanded the knowledge base on childhood obesity. The findings revealed statistically significant global clustering within the study area, along with local clustering in particular regions.

This study represents the first effort to map district-level obesity spatial clusters in the northeast of Thailand using a combination of spatial analysis techniques and obesity-related data. We found a significant, positive spatial global autocorrelation that indicated that the prevalence of childhood obesity was not randomly distributed within the study area. The global Moran I index revealed that a spatial dependence existed in the incidence of obesity among children throughout Isaan. Considering the fact that the value of this index can range from −1 to +1, indicating completely dispersed to completely clustered data, respectively, the global Moran I index obtained in this study was 0.2855. Additionally, the local Moran I index was also utilized in order to localize spatial patterns of childhood obesity within the research boundaries. In total, 12.42% of the study area was found to exhibit a high level of spatial clustering. The local Moran I analysis also revealed hot spots (HH) in major regions of the western part of Isaan, i.e., major parts in Loei, Chaiyaphum, and Nakon Ratchasima, as well as in northern regions, i.e., Bung Kan and Sakon Nakon. On the other hand, the cold spot (LL) clusters were more prominent in the southeastern part of the study area, particularly in Surin, Sri Sa Ket, and Ubon Ratchathani.

Furthermore, in this study, we examined the correlations between SES variables and childhood obesity throughout the Isaan region of Thailand. This variation was reflected in both the mapping (Figure 2) and the statistical analysis. The results of GLR revealed that the incidence of childhood obesity was positively linked with annual income, the percentage of adults with a higher education, and the percentage of unemployment, and was negatively associated with population density and the number of households. This indicated that children of a higher SES in the Isaan region exhibited a significantly higher prevalence of overweight and obesity than those belonging to a lower SES.

Our findings are statistically in line with prior research carried out in middle- and low-income countries [24,25,51]. Nevertheless, the results show remarkable asymmetry with previous studies carried out in developed countries [25,34,52,53,54]. Several studies revealed that areas with lower SES were likely to have a high prevalence of childhood obesity, e.g., those conducted in England [52], Australia [53], and in the US [55]. On the other hand, in developing countries, children living in areas of higher SES were found to be likely to have a high risk of obesity [56]. The environment in which children live and go to school can enable healthy behavior by parents and children, which helps to explain the prevalence of childhood obesity. In high-income countries, several studies indicate that poor neighborhoods frequently have unhealthy food environments [57]. Since there are fewer grocery stores in poor communities, it is more difficult for residents to obtain affordable and healthy foods, and fruit and vegetables are of poorer quality than those in more affluent neighborhoods [58,59]. However, the situation in developing countries is likely to be different to this. Energy-dense foods, such as fast food or Western-style food, are more likely to be located in urban and wealthy neighborhoods (e.g., those with higher rates of education, high income, and high employment). A recent study also revealed that in Southeast Asia the prevalence of fast-food consumption was the highest, whereas it was the lowest in the Americas [60,61]. Thailand was found to have the highest fast-food consumption—at 4–7 days per week—among other low- and middle-income countries [60]. However, rice and vegetable dishes have traditionally formed the basis of Thai cuisine, which has indeed been found to be relatively low in fat. However, over several generations, Thai food culture is rapidly changing. The increased consumption of oil, animal fats, and protein and the decreased consumption of vegetables and fruit are factors contributing to Thailand’s obesity problem. Another study carried out in North Eastern Thailand also revealed that the most popular foods consumed by young people were deep-fried chicken and hot dogs, followed by sandwiches, donuts, hamburgers, French fries, and pizza [62]. Additionally, previous research also revealed that Western diets were more likely to be consumed in overweight neighborhoods compared to areas with normal and underweight residents [63]. On the other hand, the unavailability of fast food and Western-style nutrition in areas of a low economic status may be one reason why the lower-SES areas are being protected against obesity.

In our analysis, the variable that was the most highly correlated with childhood obesity was the education level. This variable was identified as the most significant predictor of childhood obesity in the study area by both the GLR and GWR models. This finding is consistent with previous research conducted in other countries of lower economic status [5,64,65,66]. A positive correlation between obesity and parents’ education was found in Croatia [64,66], Sub-Saharan Africa [5], Kenya, and Cambodia [65]. This could be explained due to social norms in some developing countries, where a more overweight child is perceived as a healthy child, with adequate food and food security, especially among families in higher-SES communities in poor countries. Several studies also revealed that the role of tradition and parental perception have a significant impact on the health status of children. Most mothers in middle-income countries perceived that other family members would be concerned and criticized if their child was slim [67,68]. A thin child was more likely to be perceived as coming from a poor family by others in Bangladesh [68]. This perception appears to be deeply embedded in South Asian societies, as this region of the world is well known for having high rates of undernutrition [69]. Therefore, most mothers in several studies perceived childhood overweight/obesity as a sign of good health [68,70].

Furthermore, our results also revealed that there was an inverse relationship between childhood obesity and both the population density and number of households. In developing countries, a low population density and a small number of households are associated with agricultural areas or rural areas, compared to the high population density and numerous households found in urban areas. Our study area, Isaan, is dominated by agriculture and farming. The majority of the nation’s poor are concentrated in this region and it has suffered from under-investment and infrastructure [71]. This also explains the relationships observed between living conditions, in which most people might not have access to healthy food, which has become a critical differentiator between the urban and rural areas. Healthy low-calorie foods such as whole grains and brown rice are likely to be more costly than the most commonly consumed and more energy-dense diet (e.g., sticky rice and white rice). Combined with a lack of knowledge about the health risks associated with being overweight, this may explain the positive relationship between environmental education levels and child obesity in low-income countries.

Methodologically, the GWR model in this study proved to be an effective technique for analysis. GWR revealed more about the associations between childhood obesity and SES factors than the GLR model alone. With better performance, indicated by a lower AICc, the GWR model predicted a higher percentage of deviance, signifying that the local regression model performed better than the global model. Significant local clusters were highlighted on the map, with spatial connections among high and low obesity rates; therefore, these results could support health practitioners, communities, or authorities in intervening and targeting more resources towards these high–high regions. According to our GWR findings, the relationship between influential factors and the prevalence of obesity in this HH region was area-specific. In the western and northern parts of Isaan, this study demonstrated that children living in high-SES areas were more likely to be obese. Due to urbanization, which improves access to education, employment, and income, as well as the increased availability of a variety of foods, diets rich in saturated and sugars and increased sedentary behavior, which were more accessible to and/or affordable for those of higher SES, could explain the outcomes of this study. As a region-specific suggestion, the mitigation of childhood obesity could be included in urban planning, and campaigns could be developed to enhance knowledge more in these regions. To effectively reduce spatial disparities in the prevalence of childhood obesity, authorities could minimize SES disparities at the district level, e.g., by promoting access to supermarkets and low-calorie local food campaigns, as well as enhancing the awareness of obesity risks for children.

There are some limitations to this study. First, this was a cross-sectional study, and causality cannot be attributed to the findings. This has also been a limitation for several other health-related spatial clustering studies. Thus, further research in the form of longitudinal studies analyzing obesity patterns in terms of space and time are required in order to fully understand whether the spatial pattern of childhood obesity persists over time and in other communities. Second, several factors were not considered in this present research, such as behavioral factors, genetic factors, and built environmental factors due to the lack of relevant data. Therefore, additional factors, including fat, sugar, fruit and vegetable consumption, breastfeeding, parents’ obesity and behavior, and the presence of park and green areas, could be included in future research to improve and find out more significant predictors of childhood obesity. Third, as one of the aims of this study was to investigate spatial inequalities to further enhance the policies and interventions by local authorities in this area, districts were used as the administrative unit in this study. Nevertheless, the area-based methods and the district-level analysis applied in this study may have led to inconsistencies related to the modifiable areal unit problem (MAUP) due to the various sizes of districts and population distributions within each area. In this study, the size of each district varied between 54.1 and 2134.5 square kilometers, with an average area of 519.5 square kilometers, and districts had populations ranging widely between 7204 and 243,012, with an average of 45,518 people in each district. These population data were aggregated on the basis of selection in this analysis as a district boundary. However, when changing spatial zones such as provinces or other administrative units, MAUP can impact the outcomes, as well as visual and statistical results. The relationships examined as part of the analysis could be different depending on the scale of observation and the extent of the study area. In future works, the analysis could be extended by applying the population-weighted approach and expanding the analysis to other regions or scales, addressing the MAUP as well as the heterogeneity of both SES and childhood obesity. Lastly, in this study, we used secondary data that were officially collected by the Ministry of Public Health, Thailand, and published as an open database for any purpose, including research and further analysis. The ministry is responsible for monitoring and regulating the obesity situation among children in the country therefore, it was this organization that formally collected the obesity data from children at the administrative level. Regarding the validation of these data for the potential presence of bias, further investigations of the actual data collection processes used in these areas could be undertaken for a more specific and comprehensive consideration of the results.

## 5. Conclusions

In this study, we explored the spatial distribution of childhood obesity and the relationship between SES factors across the Isaan region of Thailand, a region with such geographical disparities. The results of this study highlighted the global and local spatial patterns of obesity in children aged less than 5 years old, which emphasized the significance of spatial relationships between health conditions, particularly obesity. The empirical outcomes showed that spatial autocorrelation in the prevalence of childhood obesity revealed the presence of spatial heterogeneity across Isaan. Significantly, high–high clusters of childhood obesity were found in several districts of the western part of Isaan, as well as in northern regions. Furthermore, a large concentration of low-obesity clusters were observed among children in the southeastern areas of Isaan. Furthermore, SES characteristics measured at the district level were identified and found to be associated with the prevalence of childhood obesity in both the global and local statistical approaches. High annual income, a larger proportion of higher-educated residents, and a higher rate of unemployment were statistically significantly and positively associated with the prevalence of childhood obesity, whereas population density and the number of households were negatively related to childhood obesity over space in both models. Our findings also highlight the potential of using a GIS-based approach with spatial analysis, based on data availability, that can be extended to other studies using smaller geographic units in exploring and supporting effective local policy implementation or decision-making for obesity intervention. Indeed, more research is required to investigate other possible factors that may impact the trends of childhood obesity in a local context due to the widening of economic growth in Thailand.

## Figures and Tables

**Table 1:** Data collected and used in this study.

Data Information	Source	Geographical Level	Year	Data Type
Childhood obesity data	Health Data Center, Ministry of Public Health Thailand	District level	2019	CSV file
Demographic data	Community Development Department, Thailand	District level	2019	CSV file
GIS-boundary data	Humanitarian Data Exchange (HDX)	Province level, District level	2019	Shape file

**Table 2:** Statistical results of the variables in the study.

Information	Mean	Min	Max	SD
Childhood obesity index	111.42	34.14	230.47	33.88
Annual uncome (Thousand THB)	69.64	47.61	147.64	11.59
Population density (person/sqkm)	93.20	13.17	297.56	37.06
Household (thousand households)	14.75	2.17	107.68	11.86
Percentage of adults with higher education	6.20	2.83	21.26	2.32
Percentage of unemployment	4.50	1.39	9.22	1.39
Percentage of immigrants	0.25	0.00	6.46	0.56

**Table 3:** Global Moran I and local Moran I results.

	Global Moran I ***		Local Moran I *			
	Index Value	Z-Score	HH (No., %)	HL (No., %)	LH (No., %)	LL (No., %)
Isaan	0.2855	15.58	40 (12.42%)	7 (2.17%)	8 (2.48%)	52 (16.15%)

* *p* < 0.05, *** *p* < 0.001.

**Table 4:** Results of the GLR analysis of the relationship between the SES variables and childhood obesity.

SES-Variables	GLR Results			
	β	ε	*p*-Value	VIF
Annual Income (thousand THB)	0.000005	0.00000	<0.001 *	1.4384
Population density (person/sqkm)	−0.002437	0.00019	<0.001 *	1.8019
Household (thousand households)	−0.000007	0.000001	<0.001 *	1.7365
Percent of adults with higher education	0.036415	0.003315	<0.001 *	2.3436
Percentage of unemployment	0.011506	0.003774	<0.001 *	1.0319
Percentage of immigrants	0.000705	0.009258	0.939321	1.0176

* *p* < 0.01.

**Table 5:** Results from GLR analysis (Global) and GWR analysis (Local).

Model	AICc	Deviance Explained by the Model
Global model	4904.0	0.1621
Local model	1835.8	0.8844

**Table 6:** Summary of the coefficients of the significant variables measured by GWR.

SES-Variables	Mean	Std	Median
Annual income (thousand THB)	0.0000025	0.0000071	0.0000020
Population density (person/sqkm)	−0.0013418	0.0021574	−0.0014000
Household (thousand households)	−0.0000086	0.0000058	−0.0000070
Percent of adults with higher education	0.0424146	0.0393305	0.0430600
Percentage of unemployment	−0.0151949	0.0440473	−0.0029275

**Table 7:** Mean coefficient values measured at the district level in relation to childhood obesity clusters in Isaan.

Cluster	Annual Income	Unemployment Rate	Population Density	Household	High Education Rate
HH	0.0000019	0.0138365	−0.0008796	−0.0000100	0.0712470
HL	−0.0000009	−0.0354249	−0.0029119	−0.0000061	0.0405993
LH	0.0000008	0.0409460	−0.0008311	−0.0000065	0.1306439
LL	0.0000000	−0.0337216	−0.0018572	−0.0000010	0.0536232

## Data Availability

The data presented in this study are available on request from the corresponding author.
